# Factors Associated to Prevalence and Incidence of Carbapenem-Resistant *Enterobacteriaceae* Fecal Carriage: A Cohort Study in a Mexican Tertiary Care Hospital

**DOI:** 10.1371/journal.pone.0139883

**Published:** 2015-10-02

**Authors:** Pedro Torres-Gonzalez, Miguel Enrique Cervera-Hernandez, María Dolores Niembro-Ortega, Francisco Leal-Vega, Luis Pablo Cruz-Hervert, Lourdes García-García, Arturo Galindo-Fraga, Areli Martinez-Gamboa, Miriam Bobadilla-del Valle, Jose Sifuentes-Osornio, Alfredo Ponce-de-Leon

**Affiliations:** 1 Department of Infectious Diseases, Laboratory of Clinical Microbiology, Instituto Nacional de Ciencias Médicas y Nutrición Salvador Zubirán, Mexico City, Mexico; 2 Centro de Investigación sobre Enfermedades Infecciosas, Instituto Nacional de Salud Pública, Cuernavaca, Morelos, Mexico; 3 Department of Medicine, Division of Hospital Epidemiology, Instituto Nacional de Ciencias Médicas y Nutrición Salvador Zubirán, Mexico City, Mexico; 4 Department of Medicine, Instituto Nacional de Ciencias Médicas y Nutrición Salvador Zubirán, Mexico City, Mexico; University of Calgary, CANADA

## Abstract

**Background:**

Carbapenem-resistant *Enterobacteriaceae* (CRE) infections have emerged as a serious threat to health worldwide. They are associated with increased morbidity and mortality and are capable of silently colonizing the gastrointestinal tract. Because of this, there is great interest to characterize the epidemiology of CRE carriage and acquisition in healthcare facilities. The aim of this study was to determine the prevalence and factors associated with CRE fecal carriage (CRE-fc), and risk factors for incident cases.

**Methods/Results:**

A cohort study was conducted at a tertiary care hospital from January 1^st^ to April 30^th^, 2014 during a CRE outbreak. Weekly rectal swabs were performed in patients considered at risk until discharge. CRE-fc prevalence was 10.9% (CI 95% 7.7–14.7) among 330 patients. Treatment with carbapenems (OR 2.54, CI 95% 1.15–5.62); transfer from an institution (OR 2.16, CI 95% 1.02–4.59); multi-drug resistant infection within the previous six months (OR 2.81, CI 95% 1.47–5.36); intensive care unit admission (OR 0.42, CI 95% 0.20–0.88); hematologic malignancy (OR 4.02, CI 95% 1.88–8.06); invasive procedures (OR 2.18, CI 95% 1.10–4.32); and sharing a room with a known CRE carrier (OR 3.0, CI 95% 1.43–6.31) were independently associated factors for CRE-fc. Risk factors associated with CRE-fc incidence were determined for 87 patients initially negative and with subsequent screening; the incidence rate was 2.5 cases, per 1000 person-years (CI 95% 1.5–3.9). Independently associated risk factors were carbapenem treatment (HR 2.68, CI 95% 1.03–6.98), hematologic malignancy (HR 5.74, 95% CI 2.46–13.4) and a mean daily colonization pressure ≥10% (HR 5.03, IC 95% 1.77–14.28). OXA-48-like (OXA-232) and CTX-M-15 were the predominantly identified mechanisms of resistance.

**Conclusions:**

We found an elevated incidence and prevalence of CRE-fc in our hospital. Hematologic patients need to be considered a population at risk, and antibiotic stewardship along with infection control programs need to be improved to avoid nosocomial spread.

## Introduction

Carbapenem-resistant *Enterobacteriaceae* (CRE) have emerged as a cause of nosocomial infections in several regions around the world. Because of their progressive geographic dissemination and limited therapeutic alternatives, they are now considered an important public health threat classified as urgent by the Centers for Disease Control and Prevention.[1,2] Asymptomatic colonization of the gastrointestinal tract by CRE may precede infection, and this constitutes a reservoir for transmission that may remain unidentified in hospitals that do not implement active surveillance testing.[3,4] Factors associated with CRE fecal carriage (CRE-fc) include antibiotic exposure, malignancy, non-surgical invasive procedures, prolonged hospital stay, admission to intensive care units (ICU), and sharing a room with known carriers.[3,5–7] However, most studies are retrospective and were conducted in areas with high prevalence of *Klebsiella pneumoniae* carbapenemase (KPC) or New Dehli Metallo-β-lactamase (NDM) carrying strains; therefore, these findings may not apply to other settings.[4]

Since the second half of 2012, we have observed a steady increase in CRE clinical isolates at our institution. Additionally, in December 2013, we detected an outbreak at the ICU caused by a clone of *K*. *pneumoniae* harboring an NDM-1 β-lactamase. In response to this, a set of infection control measures was implemented throughout the hospital. The aim of this study was to determine the factors associated with the prevalence and incidence of CRE-fc within the acute hospital setting.

## Methods

### Patients and Setting

A cohort study was conducted at a 230-bed tertiary care hospital in Mexico City. Subjects were adults (≥18 years old) admitted between January 1^st^ and April 30^th^, 2014, presenting with any of the following criteria: admission to the ICU, abdominal sepsis diagnosis (evidence of inflammatory systemic response in patients with previous intra-abdominal surgery and/or hollow viscera perforation and/or image showing intra-abdominal abscess), transfer from an institution (as opposed to admission from home), solid organ or bone marrow transplant within the previous six months, CRE infection, or sharing a room or unit for ≥ 48 hours with a known CRE-infected or carrier patient. From December 2013, CRE-fc screening upon admission to the ICU and, for the patients outside the ICU as soon as a patient was identified meeting any of the above criteria. The CRE-fc screening was established as a mandatory standard of care intervention by the Institutional Infection Control Committee. Because of this, a waiver for informed consent for CRE-fc screening and data collection was sought and granted by the Institutional Ethics Review Board (Comité Institucional de Investigación Biomédica en Humanos [reference 1523]). Authors involved in data analysis and manuscript preparation could not identify individual patients as the database used had a different set of identification numbers specific to this study.

### Infection Control Measures, Screening, Definitions, and Data Collection

Within 48 hours of ICU admission, or at the time the patients were identified as meeting any other eligibility criteria, an initial rectal swab (RS), for CRE-fc screening, was performed to all subjects and repeated weekly until positive or discharge from the ICU (provided that no additional criteria other than ICU admission remained present) or until discharge from the hospital.

Prior to the outbreak, there was no standardized infection control protocol in place for patients identified harboring CRE. Infection control measures were implemented for all patients included in this study from day one as follows: upon identifying a positive RS, Infection Control staff was alerted, and patients were placed in contact precautions, which included the use of disposable gowns, latex gloves and the use of special medical instruments for each patient. Additionally, cohorting of the CRE fecal carriers was performed as follows: patients sharing a multiple-bed room were moved to a single-bed room. Whenever this was not possible, CRE-fc patients were placed in designated rooms where everyone was colonized. Hand hygiene and environmental sanitation were reinforced in all hospital areas, especially in the ICU.

Patients were followed during their hospital stay; demographic and clinical information was collected upon entry and weekly, until last screening, using a previously validated questionnaire. For each study day and each shared unit in the hospital, the point prevalence of CRE was calculated as follows: number of known patients colonized or infected by CRE divided by the number of occupied beds in the unit. Next, for each patient determined to be negative on admission, the average point prevalence of CRE from admission to the first positive or last negative screening was calculated (“Colonization Pressure”). This was determined for ICU and no-ICU patients as well. Antibiotic exposure was considered as present with even a single dose of antibiotic administered. The Charlson weighted index was used to assess the level of underlying comorbidity.[8] This index was dichotomized at ≥3 to indicate a high degree of comorbidity, as suggested by previous studies.[7,8]

History of infection by multi-drug resistant organisms (MDRO) cultured from any anatomical site within the previous six months (excluding CRE infections) was assessed using the Clinical Microbiology Laboratory´s electronic database. [9,10] Invasive procedures were documented, including bronchoscopy, digestive tract endoscopy, and invasive mechanical ventilation. The presence of invasive medical devices was documented as well, including central venous catheters, or urinary catheters for a minimum of 24 hours.

Hospital admissions during the study period were retrospectively examined to identify patients who met inclusion criteria but were not included. Their medical records were reviewed, and key data was obtained for comparison with study participants to assess for bias.

### Microbiology Procedures

After the RS had been performed, samples were inoculated immediately in trypticase soy broth (5mL) with a 10μg ertapenem disk (Oxoid, Basingstoke Hampshire, England), and processed as previously described.[11] Bacterial colonies were identified and tested for antimicrobial susceptibility using the Vitek 2 system (bioMerieux, Durham, NC, USA) and Clinical and Laboratory Standards Institute interpretive criteria. [12] Additionally, the disk diffusion test was performed for ertapenem, imipenem, and meropenem. Patients were determined positive for CRE-fc if the isolate was resistant to any carbapenem (ertapenem, imipenem or meropenem). Molecular detection of β-lactamases was performed by PCR (*bla*_IMI_, *bla*_VIM_, *bla*_NDM,_ *bla*_GES_ *bla*_CTX-M-15_, *bla*_OXA-48-like,_ *bla*_SHV_, *bla*_TEM,_ *bla*_KPC,_ and *bla*_IMP_). Additionally, multiplex PCR was used to detect AmpC β-lactamases using previously described primers.[13,14] PCR-generated fragments were purified by Qiaquick PCR purification spin columns (Qiagen, Venlo, Netherlands), and sequenced with a Genetic Analyzer 3130 xL automated sequencer (AB Applied Biosystems, Hitachi, San Francisco, USA). The nucleotide sequences were analyzed at the National Center for Biotechnology Information website (http://www.ncbi.nlm.nih.gov).

### Statistical Methods

Bivariate and multivariate analyses were used to test for differences between patients with and without CRE-fc using two approaches: for the first approach prevalence analysis was performed including all study participants with CRE-fc positivity on the first or subsequent RS as the dependent variable. Characteristics of participants were compared according to CRE-fc using Student’s unpaired t-test or Mann-Whitney U test for variables for continuous variables. Categorical variables were tested by Chi-squared test. The association between CRE-fc prevalence and demographic and clinical variables was evaluated using logistic regression analysis. The variables included in the models were those with *p*-values ≤0.20 in bivariate analysis or with biological plausibility. Covariates were arrived to by using hierarchical backward elimination approach. The logistic regression models were validated by evaluating the goodness of fit, model specificity and multicollinearity. We estimated the odds ratios (OR) and 95 percent confidence intervals (CI). In a second approach, an incidence analysis was performed including patients with more than one RS and whose first RS was negative. We estimated adjusted hazard ratios (HR) and 95% CI using Cox proportional hazards models to assess the association of demographic and clinical variables with CRE-fc. In these models, the outcome was the time to positivity from admission to the first CRE- positive RS or the last negative. The proportional hazards assumption was verified by introducing terms for the interaction between time and covariates into the model. As with regression models, variables included in the models were those with *p*-values ≤0.20 in bivariate analysis or with biological plausibility and covariates were arrived to by using a hierarchical backward elimination approach.

Additionally, we compared the characteristics of study participants and eligible non-participants. All statistical analyzes were performed using STATA 11.0 software (StataCorp, College Station, TX, USA).

## Results

**Fig 1:**
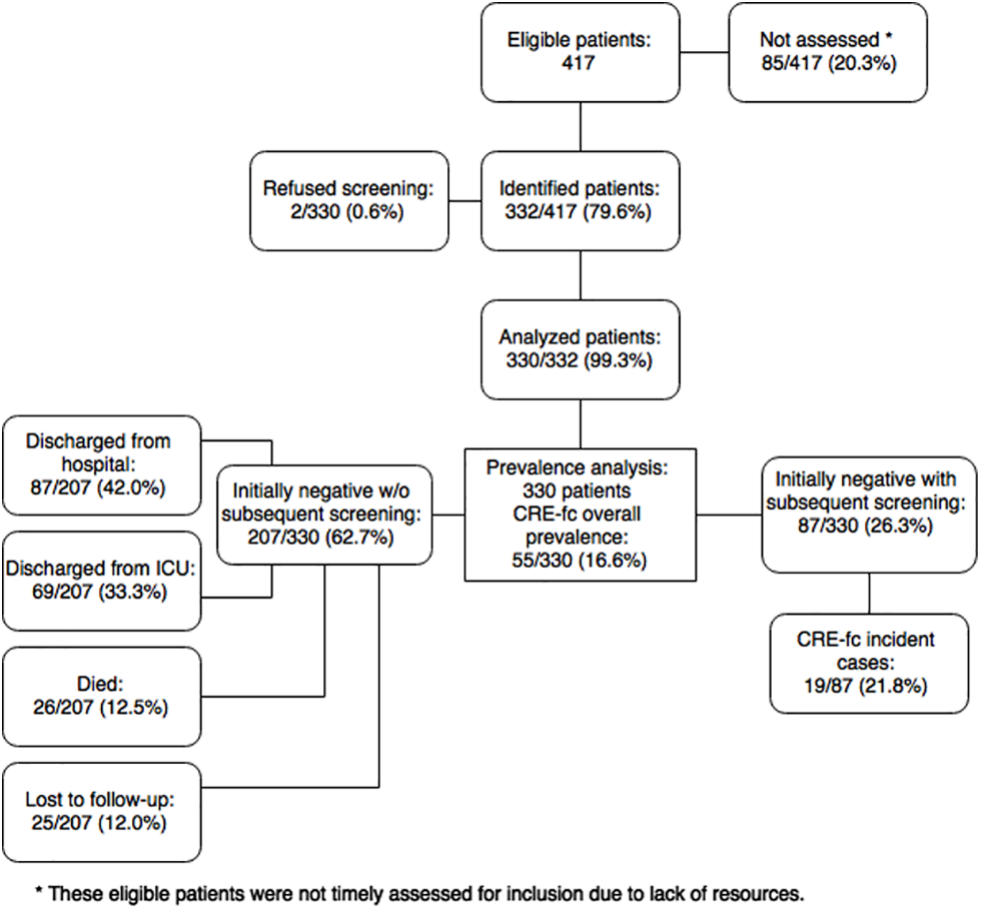
Study flowchart. CRE-fc, Carbapenem-resistant *Enterobacteriaceae* fecal carriage.

Of the eligible population (417), 330 (79.1%) participated with two patients refusing (Fig 1). One hundred and ninety were ICU patients (57.6%). Reasons for non-inclusion of the rest of the eligible patients were a lack of personnel or resources to collect RS or lack of timely information on patients meeting eligibility criteria before death or hospital discharge. We compared characteristics of participating and non-participating patients. Non-included patients were more likely to have been diagnosed with abdominal sepsis, and to have been treated with carbapenems; and less likely to have been admitted to the ICU, and to harbor gastrointestinal and pancreatobiliary disease (Table 1).

**Table 1:** Analysis of clinical and demographical differences among participants and non-participants.

Characteristic	Total n/N (%)	Eligible non-participants n/N (%)	Study participants n/N (%)	p^c^
	N = 415	n = 85	n = 330	
**Male sex**	181/415 (43.6)	35/85 (41.2)	146/330 (44.2)	0.611
**Age, years, median (IQR)**	49 (32–64)	52 (32–66)	49 (32–64)	0.569^d^
**Charlson comorbidity index ≥3**	177/415 (42.6)	40/85 (47.0)	137/330 (41.5)	0.356
**Transplant**	55/415 (13.3)	13/85 (15.3)	42/330 (12.7)	0.533
**Cancer**	66/415 (15.9)	14/85 (16.5)	52/330 (15.8)	0.873
**Rheumatologic disease** ^**a**^	55/415 (13.3)	13/85 (15.3)	42/330 (12.7)	0.534
**Hematologic malignancy**	53/415 (12.7)	16/85 (18.8)	37/330 (11.2)	0.06
**Gastrointestinal and pancreatobiliary disease** ^**b**^	96/415 (23.1)	8/85 (9.4)	88/330 (26.7)	<0.001
**Transfer from other hospital**	73/415 (17.5)	13/85 (15.2)	60/330 (18.1)	0.532
**Abdominal sepsis diagnosis**	141/415 (34)	45/85 (52.9)	96/330 (29.1)	<0.001
**Intensive care unit admission**	225/415 (54.2)	35/85 (41.2)	190/330 (57.6)	0.007
**Surgery**	164/415 (39.5)	37/85 (43.5)	127/330 (38.5)	0.396
**Carbapenem use**	227/415 (54.7)	60/85 (70.6)	167/330 (50.6)	0.001
**Vancomycin use**	255/415 (61.4)	60/85 (70.6)	195/330 (59.0)	0.052

IQR, interquartile range.

^a^ Systemic lupus erythematosus, rheumatoid arthritis, Sjögren syndrome, vasculitis.

^b^ Chronic or acute pancreatitis, inflammatory bowel disease, gastrointestinal hemorrhage, diverticulitis, short bowel syndrome, acute or recurrent cholangitis, benign biliary disease.

^c^ Chi-squared test.

^d^ t-test.

The overall prevalence of CRE-fc was 16.6% (55/330; CI 95% 12.8–21.1). Among these, 36 (65.4%) were positive on the first RS; 13 of them (23.6%) had already a documented infection by a CRE: blood (n = 4), urine (n = 4), sputum (n = 3), and intra-abdominal abscess (n = 2). Ninety-seven patients (29.9%) were screened within the first 48 hours of hospital stay; of these, 8.2% (8/97) were determined as positive for CRE-fc. Among the 294 patients whose initial RS was negative, we were able to collect more than one RS in 87 patients documenting 19 incident cases (Fig 1). Reasons for not procuring a subsequent RS included death, discharge from ICU or the hospital before the date of the next RS, or loss to follow-up. Among the 87 follow-up patients, the median time between admission and CRE-fc positive culture was 26 days (Interquartile range [IQR] 12–46). The incidence rate was 2.5 cases, per 1,000 person-years (CI 95% 1.5–3.9).

### Factors Associated to CRE-fc Prevalence

By bivariate analysis, CRE carriers were more likely to be male, younger, transplant recipients, with hematologic malignancy, history of MDRO infection, and to have been transferred from other institution. In addition, CRE-fc was associated with various healthcare-related events: hospital-acquired pneumonia; prolonged hospital stay; invasive procedures; treatment with carbapenems, vancomycin, linezolid, ciprofloxacin, and piperacillin/tazobactam; and sharing a room with a known CRE carrier or infected patient (Table 2). By multivariate analysis, factors independently associated with CRE-fc included having been treated with carbapenems(Odds Ratio [OR] 2.54, CI 95% 1.15–5.62); having been transferred from other institution (OR 2.16, CI 95% 1.02–4.59); history of MDRO infection (OR 2.81, CI 95% 1.47–5.36); hematologic malignancy (OR 4.02, 95%CI 1.88–8.60); ICU admission (OR 0.42, CI 95% 0.20–0.88); invasive procedures (OR 2.18, CI 95% 1.10–4.32); and sharing a room with a known CRE carrier or infected patient (OR 3.0, CI 95% 1.43–6.31; Table 3).

**Table 2:** Characteristics Associated to Carbapenem-Resistant *Enterobacteriaceae* Fecal Carriage.

Characteristic	Total n/N (%)	CRE non-carriers n/N (%)	CRE carriers n/N (%)	p^f^
	N = 330	n = 275	n = 55	
**Male sex**	146/330 (44.2)	114/275 (41.5)	32/55 (58.2)	0.023
**Age, years, median (IQR)**	49 (32–64)	50 (32–65)	42 (31–53)	0.026^g^
**Charlson comorbidity index ≥3**	137/330 (41.5)	118/275 (42.9)	19/55 (34.5)	0.251
**Transplant**	42/330 (12.7)	40/275 (14.5)	2/55 (3.6)	0.027
**Cancer**	52/330 (15.8)	47/275 (17.1)	5/55 (9.1)	0.137
**Rheumatologic disease** ^**a**^	42/330 (12.7)	36/275 (13.1)	6/55 (10.9)	0.658
**Hematologic malignancy**	37/330 (11.2)	23/275 (8.3)	14/55 (25.4)	<0.001
**Gastrointestinal and pancreatobiliary disease** ^**b**^	88/330 (26.7)	73/275 (26.5)	15/55 (27.3)	0.911
**MDRO infection during past 6 months**	97/330 (29.4)	69/275 (25.1)	28/55 (50.9)	<0.001
**Transfer from other institution**	60/330 (18.2)	44/275 (16)	16/55 (29.1)	0.022
**Abdominal sepsis diagnosis**	96/330 (29.1)	78/275 (28.4)	18/55 (32.7)	0.515
**Hospital acquired pneumonia**	90/330 (27.3)	65/275 (23.6)	25/55 (45.5)	<0.001
**Length of stay, days, median (IQR)**	8 (3–19)	8 (3–16)	15 (8–28)	<0.001^g^
**Intensive care unit admission**	190/330 (57.6)	163/275 (59.3)	27/55 (49.1)	0.163
**Invasive procedures** ^**c**^	93/330 (28.1)	69/275 (25)	24/55 (43.6)	0.005
**Invasive medical devices** ^**d**^	278/330 (84.2)	230/275 (83.6)	48/55 (87.3)	0.499
**Surgery**	127/330 (38.5)	104/275 (37.8)	23/55 (41.8)	0.578
**Surgical drain**	124/330 (37.6)	101/275 (36.7)	23/55 (41.8)	0.477
**Enteral tube feeding**	104/330 (31.5)	84/275 (30.5)	20/55 (36.3)	0.396
**Percutaneous abdominal drainage**	30/330 (9.1)	25/275 (9.1)	5/55 (9.1)	1.000
**Pharmacologic immunosuppression during past 3 months** ^**e**^	121/330 (36.7)	100/275 (36.4)	21/55 (38.2)	0.798
**Carbapenem use**	167/330 (50.6)	127/275 (46.1)	40/55 (72.7)	<0.001
**Vancomycin (IV) use**	195/330 (59.1)	155/275 (56.4)	40/55 (72.7)	0.024
**Clindamycin use**	16/330 (4.8)	13/275 (4.7)	3/55 (5.5)	0.819
**Linezolid use**	39/330 (11.8)	25/275 (9.1)	14/55 (25.5)	<0.001
**Ciprofloxacin use**	28/330 (8.5)	18/275 (6.5)	10/55 (18.2)	0.005
**Third-generation cephalosporin use**	83/330 (25.2)	72/275 (26.2)	11/55 (20)	0.335
**Piperacillin / tazobactam use**	144/330 (43.6)	113/275 (41.1)	31/55 (56.4)	0.037
**Sharing a room with known CRE carrier or infected patient**	84/330 (25.4)	60/274 (21.9)	24/55 (43.6)	<0.001

CRE, carbapenem-resistant *Enterobacteriaceae*; IQR, interquartile range; MDRO, multi-drug resistant organism.

Data about hospitalization exposures occurred before the last screening culture.

^a^ Systemic lupus erythematosus, rheumatoid arthritis, Sjögren syndrome, vasculitis.

^b^ Chronic or acute pancreatitis, inflammatory bowel disease, gastrointestinal hemorrhage, diverticulitis, short bowel syndrome, acute or recurrent cholangitis, benign biliary disease.

^c^ Bronchoscopy, digestive tract endoscopy, or invasive mechanical ventilation.

^d^ Central venous catheter or urinary catheter (more than 24 hours).

^e^ Steroids, azathioprine, mycophenolate mofetil, tacrolimus, cyclosporine, hydroxychloroquine, methotrexate, or chemotherapy.

^f^ Chi-squared test.

^g.^ Mann–Whitney test.

**Table 3:** Characteristics Associated to Carbapenem-Resistant *Enterobacteriaceae* Fecal Carriage by Multivariate Analysis.

	Prevalence^e^ (n = 330)	Incidence^f^ (n = 87)
Characteristic	OR (95% CI)	HR (95% CI)
**Treatment with carbapenems**	2.54 (1.15–5.62)^a^	2.68 (1.03–6.98)^a^
**Treatment with vancomycin**	0.89 (0.38–2.10)	0.2 (0.02–1.41)
**Transfer from another institution**	2.16 (1.02–4.59)^a^	
**MDRO infection during past 6 months**	2.81 (1.47–5.36)^b^	
**Hematologic malignancy**	4.02 (1.88–8.60)^c^	5.74 (2.46–13.4)^c^
**Intensive care unit admission**	0.42 (0.20–0.88)^a^	0.42 (0.16–1.12)
**Invasive procedures** ^**d**^	2.18 (1.10–4.32)^a^	0.50 (0.19–1.35)
**Sharing a room with known CRE carrier or infected patient**	3.0 (1.43–6.31)^b^	
**Mean daily colonization pressure ≥ 10%**		5.03 (1.77–14.28)^b^

OR, odds ratio; CI, confidence interval; HR, hazard ratio; MDRO, multi-drug resistant organism; CRE, carbapenem-resistant *Enterobacteriaceae*.

^a^ p < .05.

^b^ p < .01.

^c^ p < .001.

^d^ Bronchoscopy, digestive tract endoscopy, or invasive mechanical ventilation.

^e^ Logistic regression.

^f^ Cox proportional hazards regression.

### Factors Associated to CRE-fc Incidence

None of the 19 incident cases of colonization developed signs or symptoms of CRE infection during their hospital stay. By bivariate analysis, incident cases were more likely to be male and to have a hematologic malignancy (Table 4). In multivariate analysis, treatment with carbapenems (Hazard ratio [HR] 2.68 CI 95% 1.03–6.98), hematologic malignancy (HR 5.74, CI 95% 2.46–13.4), and mean daily colonization pressure ≥10% (HR 5.03 CI 95% 1.77–14.28) were independently associated with CRE-fc incidence (Table 3).

**Table 4:** Characteristics Associated to Carbapenem-Resistant *Enterobacteriaceae* Fecal Carriage Incidence.

Characteristic	Total n/N (%) N = 87	CRE carriers n/N (%) n = 19	CRE non-carriers n/N (%) n = 68	p^f^
**Male sex**	29/87 (33.3)	11/19 (57.9)	18/68 (26.5)	0.042
**Age, years, median (IQR)**	49 (30–62)	45 (34–58)	50.5 (29–65)	0.837
**Charlson comorbidity index ≥3**	53/87 (60.9)	11/19 (57.8)	42/68 (61.7)	0.382
**Transplant**	5/87 (5.7)	1/19 (5.3)	4/68 (5.9)	0.709
**Cancer**	7/87 (8)	0/19 (0)	7/68 (10.2)	—-
**Rheumatologic disease** ^**a**^	16/87 (18.4)	2/19 (10.5)	14/68 (20.6)	0.284
**Hematologic malignancy**	10/87 (11.5)	7/19 (36.8)	3/68 (4.4)	<0.001
**Gastrointestinal and pancreatobiliary disease** ^**b**^	37/87 (42.5)	5/19 (26.3)	32/68 (47.1)	0.05
**MDRO infection during past 6 months**	34/87 (39.1)	10/19 (52.6)	24/68 (35.3)	0.694
**Transfer from another institution**	10/87 (11.5)	2/19 (10.5)	8/68 (11.8)	0.65
**Abdominal sepsis diagnosis**	30/87 (34.5)	7/19 (36.8)	23/68 (33.8)	0.151
**Hospital Acquired Pneumonia**	39/87 (44.8)	12/19 (63.2)	27/68 (39.7)	0.596
**Intensive care unit admission**	66/87 (75.9)	12/19 (63.2)	54/68 (79.4)	0.11
**Invasive procedures** ^**c**^	37/87 (42.5)	7/19 (36.8)	30/68 (44.1)	0.258
**Invasive medical devices** ^**d**^	79/87 (90.8)	16/19 (84.2)	63/68 (92.6)	0.263
**Surgery**	43/87 (49.4)	11/19 (57.9)	32/68 (47.1)	0.412
**Surgical drain**	40/87 (45.9)	9/19 (47.3)	31/68 (45.5)	0.189
**Enteral tube feeding**	54/87 (62.1)	11/19 (57.9)	43/68 (63.2)	0.16
**Percutaneous abdominal drainage**	16/87 (18.4)	2/19 (10.5)	14/68 (20.6)	0.214
**Pharmacologic immunosuppression during past 3 months** ^**e**^	33/87 (37.9)	8/19 (42.1)	25/68 (36.8)	0.679
**Carbapenem use**	48/87 (55.1)	11/19 (57.8)	37/68 (54.4)	0.323
**Vancomycin (IV) use**	75/87 (86.2)	17/19 (89.5)	58/68 (85.3)	0.207
**Clindamycin use**	9/87 (10.3)	2/19 (10.5)	7/68 (10.3)	0.532
**Linezolid use**	24/87 (27.6)	8/19 (42.1)	16/68 (23.5)	0.681
**Ciprofloxacin use**	15/87 (17.2)	5/19 (26.3)	10/68 (14.7)	0.317
**Third-generation cephalosporin use**	30/87 (34.5)	7/19 (36.8)	23/68 (33.8)	0.932
**Piperacilin/tazobactam use**	63/87 (72.4)	14/19 (73.7)	49/68 (72.1)	0.354
**Sharing a room with known CRE carrier or infected patient**	52/87 (59.7)	14/19 (73.6)	38/68 (55.8)	0.263
**Colonization pressure ≥ 10%**	16/87 (18.4)	5/19 (26.3)	11/68 (16.2)	0.053

CRE, carbapenem-resistant *Enterobacteriaceae*; IQR, interquartile range; MDRO, multi-drug resistant organism.

Data about hospitalization exposures occurred before the last screening culture.

^a^ Systemic lupus erythematosus, rheumatoid arthritis, Sjögren syndrome, vasculitis.

^b^ Chronic or acute pancreatitis, inflammatory bowel disease, gastrointestinal hemorrhage, diverticulitis, short bowel syndrome, acute or recurrent cholangitis, benign biliary disease.

^c^ Bronchoscopy, digestive tract endoscopy, or invasive mechanical ventilation.

^d^ Central venous catheter or urinary catheter (more than 24 hours).

^e^ Steroids, azathioprine, mycophenolate mofetil, tacrolimus, cyclosporine, hydroxychloroquine, methotrexate, or chemotherapy.

^f^ Cox proportional hazards regression.

### Microbiological Features

Overall, there were 55 cases of CRE-fc. Forty (72.7%) were identified as *Escherichia coli*, 13 (23.6%) were *K*. *pneumoniae*, and two (3.6%) were *Enterobacter cloacae*. Molecular mechanisms of resistance to carbapenems were identified in 45.4% (25/55) of the isolates; the most frequent was the co-existence of OXA-232 with either a CTX-M-15 or an SHV class A β-lactamase (56%). NDM-1, KPC-1, and IMP were also identified (Table 5).

**Table 5:** Microbiology, Susceptibility Testing, and Molecular Mechanisms of Resistance of Carbapenem-Resistant *Enterobacteriaceae* Fecal Isolates.

Isolates (n = 55)	Antibiotics (μg/mL)			Resistance Mechanism (n)
Species	Ertapenem	Meropenem	Imipenem	
***Klebsiella pneumoniae* (n = 13)**				OXA-232 + CTX-M-15 (2), OXA-232 (1), KPC-1 + CTX-M-15(1), KPC-1 + SHV (1), NDM-1 + CTX-M-15 (3), Unidentified (5)
**MIC**_**50**_	8	16	16	
**MIC**_**90**_	8	16	16	
***Escherichia coli* (n = 40)**				OXA-232 + CTX-M15 (10), OXA-232 + SHV (2), OXA-232 (1), IMP + CTX-M-15 (3), IMP(1), Unidentified (23)
**MIC**_**50**_	8	2	1	
**MIC**_**90**_	8	8	2	
***Enterobacter cloacae* (n = 2)**				Unidentified (2)
**MIC**_**50**_	8	0.75	0.75	
**MIC**_**90**_	8	1	1	
**Total (n = 55)**				
**MIC**_**50**_	8	2	1	
**MIC**_**90**_	8	16	16	

MIC, minimum inhibitory concentration; MIC_50_, minimum inhibitory concentration for 50% of the given species; MIC_90_, minimum inhibitory concentration for 90% of the given species.

## Discussion

The present study shows a high incidence and prevalence of CRE-fc in a setting where the predominant resistance mechanisms are not KPC or NDM. Identification of health care- associated, and patient-associated risk factors may guide preventive and control measures in settings similar to ours, particularly in hospitals with limited resources. To our knowledge, this is the first follow-up study that has sought to identify risk factors associated with CRE-fc incidence in the region.

In the prevalence analysis, we identified transfer from other institution as an associated factor for CRE-fc. Our hospital is a referral center where patients from all over the country arrive for treatment. The majority of these patients have complex medical and surgical diseases, prolonged exposure to the healthcare setting and extensive use of broad-spectrum antibiotics. We believe this may explain the high proportion of CRE-fc among this group. Moreover, a recent study conducted in four regional general hospitals showed a 2.4% rate of CRE-fc, one-fourth the rate that we observed, and only one-fifth of their isolates produced a carbapenemase.[15] This is important mainly because their study was not associated with any particular outbreak; rather, it attests to the potential risks that general hospitals pose to tertiary care hospitals such as ours. The performance of invasive procedures was also associated with CRE-fc in our study. It has been suggested that these procedures do not represent an inherent risk for patients. Instead, they are related to disease severity or represent entry portals for potential colonizing pathogens. [16,17] We also found history of infection by MDRO within the previous six months as an associated factor in our study. This has been previously described as a measure of antibiotic exposure that may predispose to the selection of CRE in the gut.[5,6]

We found a high rate of hematologic malignancy patients with CRE-fc in both incidence and prevalence analyzes. This population is subject to constant readmission to healthcare facilities and administration of broad-spectrum antibiotics and chemotherapy agents that may disrupt the gastrointestinal microbiota, thus rendering them prone to colonization by CRE.[18,19] Mean daily colonization pressure ≥10% was also found as a risk factor for CRE-fc. This finding is supported by earlier reports of colonization by CRE and other MDRO. [3,20,21] This is a measure of CRE burden in our hospital and represents a reservoir for transmission, as suggested by our data. Along with antibiotic control, this is a potentially modifiable risk factor that highlights the importance of contact precautions and stringent hand hygiene while caring for these patients. Moreover, sharing a room with known CRE carriers or infected patients was associated with CRE-fc in the prevalence analysis. This has been previously described as an associated factor, and can be interpreted in the same manner as colonization pressure.[6]

Carbapenem treatment was identified as a risk factor for CRE-fc in both analyzes. This correlates with earlier reports [7,22] and may be explained by the effect of the selective pressure that these antibiotics exert on patients who are already colonized by CRE. There is a high rate of extended spectrum β-lactamase (ESBL) infections in our hospital, which has precipitated an increase in carbapenem prescription. We believe this is partially responsible for the appearance of CRE in recent years. We have previously reported a non-significant effect of ertapenem administration on the rise of gram-negative bacilli at our institution from 2002 to 2008; nevertheless, the occurrence of CRE began in 2012. [23] Therefore, it may well be the use of other antimicrobials or their combination that selects for this trait. Although in the present study exposure to each kind of carbapenem was analyzed separately, we did not find any difference. Since changes in the microbiota have been observed from the first antibiotic dose,it is difficult to assess the effect of a particular antibiotic within the same class.[24] We did not find an association with other antibiotics.

Contrasting with previous studies, we found that patients admitted to the ICU were less likely to be fecal carriers of CRE. An explanation for this is that the initial outbreak was detected in the ICU and active surveillance for CRE-fc was part of a bundle of actions that included patient cohorting, contact precautions, reinforcement of the hospital hygiene measures, more stringent antibiotic control, and education of health care personnel. Therefore, we believe that all of these measures were effective in diminishing the spread of CRE, especially in these areas, although no comparison period is available given that this was the first CRE outbreak at our institution.

It has been proposed that the factors associated with CRE-fc may depend on the species of *Enterobacteriaceae* and the resistance mechanism.[4,25] In our report, active surveillance was initiated in response to a *K*. *pneumoniae* NDM-1 outbreak; nevertheless, this was not the most frequent mechanism of resistance identified in the CRE isolated from RS, nor was *K*. *pneumoniae* the most common species found. Instead, we found a significant heterogeneity of CRE species and mechanisms of resistance, and in almost half of the isolates it was not possible to identify a carbapenemase. Most of these unidentified strains carried an ESBL not considered to affect carbapenem activity (CTX-M-15 or SHV); thereby, we believe that carbapenem resistance in these isolates may be explained by the combination of an ESBL with other mechanisms such as porin defects.[1] This suggests that, during the screening, we may have had detected *in vivo* development, instead of the exclusive patient-to- patient spread of CRE strains in the hospital environment. The spread potential of these strains is not as well understood as other outbreak-causing strains carrying KPC or NDM. This should be taken into consideration when interpreting the results, especially while using a high-sensitivity screening method in a setting overwhelmed by ESBL like ours.

This study has some limitations. Firstly, we were unable to study all eligible patients; therefore, our results may not be applied to patients with abdominal sepsis as shown in the analysis comparing the retrospectibly collected data of not-included patients. Secondly, carbapenem use, a previously identified as a risk factor, was more frequent among non-participants; therefore, we consider that their association with CRE-fc may have been underestimated. [7,22] Thirdly, we were not able to procure subsequent RS for a considerable amount of patients not colonized on admission. It is likely that our sensitivity to detect CRE-fc incidence would have increased if we had performed more frequent rectal swabs. Despite this limitation, our results show that the incidence of CRE-fc was high. Fourthly, we decided to include CRE-infected patients that were concurrently colonized because we believe this population contributes equally to the CRE burden in the hospital; nevertheless, this may result in an overestimation of CRE-fc prevalence. Finally, we do not have data on the prevalence previous to this study; consequently, the impact of these measures on the incidence and prevalence of CRE-fc cannot be estimated.

This study found that in the setting of high heterogeneity of mechanisms of resistance, several different factors related to the patient, the environment, and healthcare interventions are all contributing to the dynamics of CRE in the hospital. Our findings may apply to similar hospitals in low and medium resource settings, and indicate the need for reinforcing antibiotic stewardship along with infection control programs with an emphasis on patients at increased risk. The high prevalence and incidence of CRE-fc alert on the risk of dissemination to other hospitals and the community as has been previously shown for ESBL *Enterobacteriaceae*.[26]
